# Physical Activity and Sedentary Behavior on Well-Being and Self-Rated Health of Italian Public Health Medical Residents During the COVID-19 Pandemic: The PHRASI Study

**DOI:** 10.3390/sports12120332

**Published:** 2024-12-02

**Authors:** Alessandro Catalini, Giuseppa Minutolo, Marta Caminiti, Angela Ancona, Claudia Cosma, Veronica Gallinoro, Vincenza Gianfredi

**Affiliations:** 1 UOC Igiene degli Alimenti e Nutrizione, Dipartimento di Prevenzione, AST Macerata, 62100 Macerata, Italy; alecata@icloud.com; 2 Food Hygiene, Nutritional Surveillance and Prevention, Department of Prevention, Provincial Healthcare Authority of Palermo, 90129 Palermo, Italy; 3 School of Hygiene and Preventive Medicine, University of Perugia, 06100 Perugia, Italy; marta.caminiti@gmail.com; 4 School of Public Health, Vita-Salute San Raffaele University, 20132 Milan, Italy; ancona.angela@hsr.it; 5 Department of Health Science, University of Florence, 50121 Firenze, Italy; claudia.cosma@unifi.it (C.C.); veronica.gallinoro@unifi.it (V.G.); 6 Department of Biomedical Sciences for Health, University of Milan, via Pascal, 36, 20133 Milan, Italy; vincenza.gianfredi@unimi.it

**Keywords:** physical activity, well-being, self-rated health, sedentary behavior, medical residents

## Abstract

High workloads and extended work shift greatly limit the opportunities for medical residents to adopt a healthy lifestyle by practicing regular physical exercise. Using data from the Public Health Residents’ Anonymous Survey in Italy (PHRASI), this research assessed the associations between physical activity levels and sedentary behavior, well-being, and self-rated health among Italian public health residents (PHRs) during the COVID-19 pandemic. Employing a cross-sectional design, this study utilized the International Physical Activity Questionnaire, the WHO-5 Well-being Index, and the single-item self-rated health to measure physical activity, sedentary behavior, self-rated health, and well-being among PHRs. The study included 379 PHRs. Multiple logistic regressions adjusted for age and sex were applied to explore the associations among the variables of interest. While 74% of PHRs were sufficiently active, 50% reported good well-being. We found a positive association between physical activity (specifically walking and intense activities) and well-being (aOR 1.292, *p* = 0.032). At the same time, sedentary behavior was negatively associated with self-rated health (aOR 0.948, *p* = 0.022) and well-being (aOR 0.945, *p* = 0.005). This study contributes valuable insights into the role of physical activity and sedentary behavior in PHRs’ mental health, calling for targeted public health strategies to support their well-being.

## 1. Introduction

The COVID-19 pandemic has underscored healthcare professionals’ essential role in managing public health crises, with medical doctor residents in public health at the forefront [1]. These individuals have faced the dual challenges of fulfilling their educational commitments while addressing the public health crisis, highlighting concerns about their mental health and well-being due to the heightened stressors of this period [2].

During the pandemic, residents faced not only additional responsibilities but also an increased workload. The emergency situation forced many public health residents to work with minimal supervision, and some had to work in close contact with infected patients, thereby facing a higher risk of infection. These and other work-related factors greatly impacted residents’ well-being, as documented by several studies [3,4]. The pandemic has not only heightened work-related stress levels among this group, but has also led to changes in their daily routines, potentially bringing modifications to their eating patterns, changing their habits towards smoke and alcohol consumption, limiting their social interaction opportunities and increasing sedentary behaviors due to lockdowns and work-from-home directives [5]. It is well-documented that all these lifestyle determinants significantly impact mental well-being [6,7]. Furthermore, public health residents are predominantly young adults, with a higher percentage of female residents [2]. Both sex and age are well-known determinants of well-being, with evidence indicating that women and young individuals are the most vulnerable subgroups to poor well-being [8,9]. Combined with work-related stressors and lifestyle determinants, these socio-demographic characteristics represent a substantial and concerning array of factors that likely played an important role in medical residents’ overall well-being during the pandemic.

While the benefits of physical activity on mental health are well-documented, including its role in reducing symptoms of stress, depression, and anxiety [10], the impact of sedentary behavior has emerged as a significant concern, especially in the context of the pandemic [11]. However, the pandemic’s restrictions, such as longer work hours and social distancing, may have curtailed physical activity opportunities for healthcare workers [12]. Extended periods of sitting and limited physical movement are associated with adverse health outcomes, including increased risks of mental health disorders [13]. Contrariwise, doing physical activity can prevent low well-being in an emergency context like the COVID-19 pandemic [9]. The interplay between physical activity and sedentary behavior, especially in high-stress environments like healthcare settings during a pandemic, necessitates a comprehensive examination. Exploring how physical activity levels and sedentary behavior correlate with well-being and self-rated health during such crises can inform targeted interventions to support their mental health during and beyond the pandemic. While existing research has significantly explored the impact of physical activity on mental health, there is a notable gap in understanding the dual influence of physical activity and sedentary behavior, particularly among healthcare professionals, like medical residents, during crisis conditions like the COVID-19 pandemic [14,15]. This population lacks a comprehensive study of the nuanced effects of different types of physical activities (e.g., walking, moderate, and intense activities) and the distinct impacts of sedentary behaviors during weekdays and weekend days. This oversight is critical, as medical doctor residents have faced unique challenges during the pandemic, facing not only the general stressors of the healthcare profession but also the added pressures of continuous learning and adaptation to rapidly changing public health guidelines [16]. Moreover, the pandemic’s constraints on physical activity pose additional challenges to their well-being.

Thus, this study seeks to fill a critical knowledge gap by assessing not only the positive aspects of physical activity on well-being and self-rated health within Italian public health residents (PHRs), but also by focusing on the potential negative impacts of increased sedentary behavior on well-being and self-rated health. Our hypothesis was that total and different intensity levels of physical activity would improve well-being and self-rated health among PHRs during the pandemic. Conversely, sedentary behavior would reduce these outcomes. This research aims to provide results that support health promotion initiatives focused on physical activity and sedentary behaviors in a vulnerable subgroup of healthcare workers, such as PHRs, and in an emergency context like the COVID-19 pandemic.

## 2. Materials and Methods

### 2.1. Study Design and Data Collection

The Public Health Residents’ Anonymous Survey in Italy (PHRASI) is a nationwide cross-sectional study among Italian Public Health Medical Residents, who are medical doctors enrolled in a specialization school in Public Health [17]. It aims to explore various aspects of their mental health and the factors influencing it.

The study’s protocol has been previously described in detail [18]. In brief, PHRASI comprises a voluntary, anonymous, electronic survey with 88 items developed on Google Form (©2022 Google, Mountain View, CA, USA). It includes socio-demographic questions and tools for evaluating mental health and its determinants. All the questionnaires used in the survey were drawn from the existing literature and had been previously validated. The minimum required sample size was determined using the formula outlined by Charan and Biswas for cross-sectional studies, resulting in a calculated sample size of 315 [19].

Participation was voluntary, and all data were anonymous, self-reported, and collected online between 14 June and 26 July 2022. The questionnaire was implemented in Google Forms with all items as mandatory fields and disseminated through the mailing list of the medical residents’ Assembly of the Italian Society of Hygiene and Preventive Medicine. Additionally, representatives from each postgraduate Public Health school were contacted and requested to distribute the survey among their colleagues.

Ethical Committee approval was not required because the questionnaire responses were anonymized, rendering individual identification impossible. Data analysis was conducted solely in aggregate form and in compliance with relevant Italian and European regulations governing personal data management [20,21,22].

For the current study, employing a cross-sectional design, the analysis was performed using socio-demographics data and information collected from the International Physical Activity Questionnaire, the single-item self-rated health, and the WHO-5 Well-being Index to measure physical activity and sedentary behavior, self-rated health, and well-being among PHRs.

### 2.2. Socio-Demographic Variables

The socio-demographic characteristics included in the analysis were as follows: age, sex, region of residence, region of work, cohabitation status, and relationship status.

Age was treated as a continuous variable. Region of residence and region of work were classified into “North”, “Center” and “South and Islands” according to the Italian National Institute of Statistics (ISTAT). “North” includes the following regions: Aosta Valley, Liguria, Lombardy, Piedmont, Emilia-Romagna, Friuli-Venezia Giulia, Trentino-Alto Adige/Südtirol, and Veneto; “Center” includes Lazio, Marche, Tuscany, and Umbria; and “South and Islands” includes Abruzzo, Apulia, Basilicata, Calabria, Campania, Molise, Sardinia, and Sicily. The remaining variables were dichotomized as follows: cohabitation was divided into “people who live alone” or “live with others”, regardless of whether they lived with roommates or family members; having a stable relationship was dichotomized as “No” or “Yes”.

### 2.3. Physical Activity and Sedentary Behavior

Physical activity levels were measured using the short form of the International Physical Activity Questionnaire (IPAQ) [23,24]. The IPAQ is a validated tool designed to assess participants’ engagement in physical activity over the past seven days. It solicits information on the frequency (days per week) and duration (minutes per day) of vigorous and moderate activities, as well as walking. Additionally, participants are asked to report the time (in minutes) spent sitting on both weekdays and weekend days. To facilitate our analysis, we computed the mean daily duration of vigorous, moderate, and walking activities by multiplying frequency and duration for each physical activity level and dividing by seven (days of the week). Subsequently, we aggregated the durations of these activities to derive a variable representing total daily physical activity. Similarly, we summed the reported durations of sitting on weekdays and weekend days to generate a variable representing total sitting time. For clarity in presenting results, all physical activity variables were expressed in minutes per day during descriptive analysis. However, for logistic regressions, we converted these variables to hours per day to enhance readability. Furthermore, in order to compare our data to an internationally recognized benchmark and facilitate comparisons with results from other population groups, we assessed the PHRs’ compliance with the WHO physical activity guidelines [25]. For this purpose, we performed additional analyses, using the information about moderate and vigorous physical activity retrieved from the IPAQ to generate two dichotomous variables representing the adherence to the recommended weekly amount of moderate and vigorous activity.

### 2.4. Well-Being

The 5-item Well-being Index (WHO-5) was used to assess the well-being of participants [26,27]. The WHO-5 has been widely used in research to evaluate psychological well-being and has been validated in various populations, including healthcare professionals. This questionnaire comprises five items, each rated on a 6-point Likert scale, where higher scores indicate better well-being. The final score varies between 0 and 25, with 0 indicating the lowest level and 25 indicating the highest level of well-being. For our analysis and following WHO’s approach, we defined a respondent’s well-being as “high” if WHO-5 ≥ 13 and “low” if WHO-5 < 13 [28]. The Cronbach’s alpha for the WHO-5 calculated on the PHRASI sample was 0.85 (95% CI = 0.82–0.87).

### 2.5. Self-Rated Health

The self-rated health (SRH) is a concise tool consisting of the following single question “In general, how would you rate your health?” [29,30]. Possible answers are “excellent”, “very good”, “good”, “fair” and “poor”. This instrument provides a brief evaluation of the respondent’s overall perceived health. For the purpose of this analysis, the possible responses were grouped into “high” (“excellent” to “good”) and “low” (“fair” and “poor”). SRH is a powerful predictor of future health outcomes, capturing essential aspects of health that are not fully measured by objective indicators [31]. Its validity is well supported across various populations due to its robustness and reliability across different cultural and demographic groups [32].

### 2.6. Statistical Analysis

Descriptive statistics were used to summarize participants’ demographics, physical activity levels, sedentary behavior, well-being scores, and self-rated health. Categorical variables were reported in absolute and percentage relative frequencies, whereas mean and standard deviation (SD) were calculated for continuous variables. The Wilcoxon rank sum test was chosen to evaluate the distribution of the continuous variables representing different levels of physical activity and sedentary behavior according to different groups of well-being and self-rated health.

Multivariate logistic regression analysis adjusted for the possible confounders, age and sex, was performed to evaluate the associations of physical activity levels and sedentary behavior with well-being and self-rated health [33]. The reason behind the adjustment for age and sex is the great influence of these factors in the development of mental conditions that affect well-being as is well documented in the literature [34].

To explore the compliance with the WHO physical activity guidelines [25], an additional analysis was performed considering the two dichotomous variables that measure the achievement of the recommended moderate and intense weekly exercise. The chi-square test or Fisher’s exact test, when appropriate, was used to explore the distribution of adherence to WHO guidelines across different levels of well-being and self-rated health [25]. Subsequently, another logistic regression analysis adjusted for sex and age assessed the association of the adherence to recommended weekly physical activity with well-being and, secondly, with self-rated health [25]. Adjusted odds ratio (aOR) and related 95% confidence interval (95% CI) were reported for all included variables in the models.

All results were considered statistically significant whether the *p*-value was below 0.05. The statistical software for all the analysis was R 4.3.2 [35].

## 3. Results

### 3.1. Participants’ Demographics

**Figure 1:**
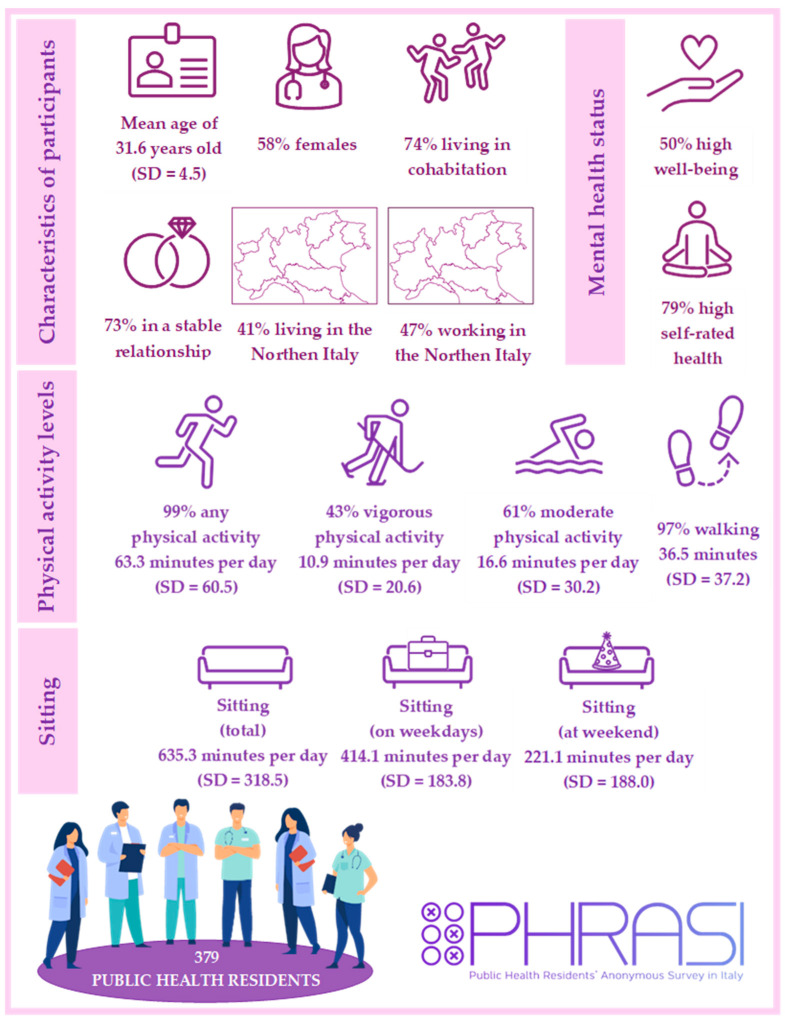
Socio-demographic characteristics and physical activity levels of PHRs. Icons on moderate and vigorous physical activity were chosen according to Compendium of Physical Activities (https://pacompendium.com/sports/ (accessed on 22 November 2024)), considering the metabolic equivalent of task (MET) thresholds of physical activity levels reported by Haskell et al. (https://journals.lww.com/acsm-msse/fulltext/2007/08000/physical_activity_and_public_health__updated.27.aspx (22 November 2024)). Some of the graphical elements are 100% free images by pch.vector on Freepick.

A total of 379 PHRs participated in the study. The mean age of the participants was 31.6 years (SD = 4.5), with 58% being female. In total, 47% of the sample worked in the North of Italy, 30% in the Center, and 23% in the South and the Islands. In total, 74% of the participants lived with other people and 73% were in a stable relationship. Detailed and further demographic information, including geographic residence, is presented in Figure 1 and in Table S1. Regarding SRH, the range was from 1 to 5, with a mean of 3.2 (SD = 0.96). The WHO-5 ranged from 1 to 25, with a mean score of 12.2 (SD = 4.6). Half (*n* = 188) of the participants declared a high level of well-being.

### 3.2. Levels of Physical Activity and Sedentary Behavior

Almost all the participants performed any level of physical activity (99%, *n* = 361). In detail, analysis of the IPAQ responses indicated that 43% of the participants engaged in vigorous levels of PA, 61% in moderate PA, and 97% in walking (Figure 1).

**Figure 2:**
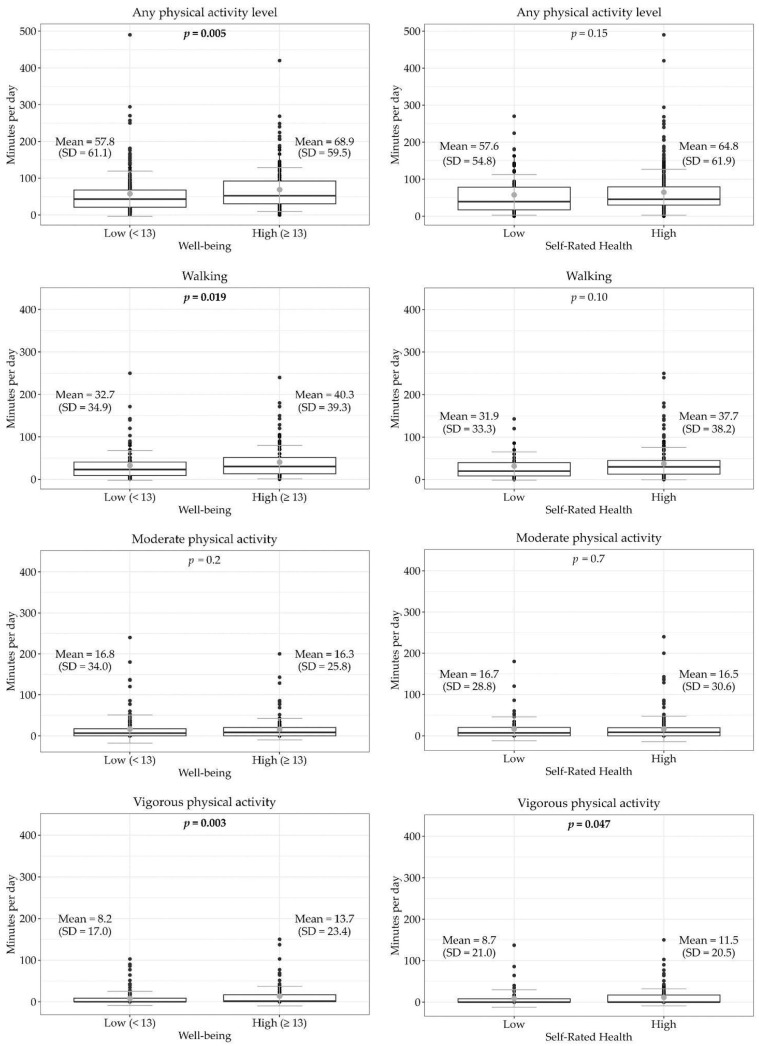
Boxplots showing the mean (gray dot) and SD (gray line) of minutes per day spent on different levels of physical activity, grouped by well-being and self-rated health.

**Figure 3:**
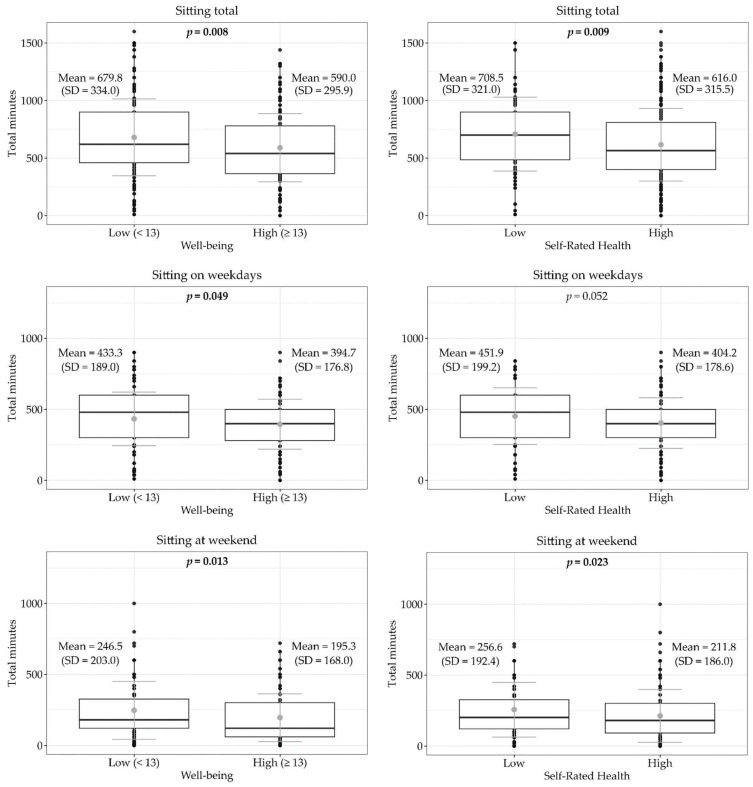
Boxplots showing the mean (gray dot) and SD (gray line) of minutes spent in sedentary behavior (weekly, on weekdays, and at the weekend), grouped by well-being and self-rated health.

In Figure 2, distributions of physical activity levels and sedentary behavior (Figure 3) were reported according to well-being and SRH. Participants with higher well-being (WHO-5 ≥ 13) spent more minutes per day on any physical activity (68.9 vs. 57.8, *p* = 0.005), walking (40.3 vs. 32.7, *p* = 0.019), and vigorous physical activity (13.7 vs. 8.2, *p* = 0.003). In contrast, participants with lower well-being (WHO-5 < 13) showed higher sitting time both considered as a total (679.8 vs. 590.0, *p* = 0.008) and separately for week (433.3 vs. 394.7, *p* = 0.049) and weekend days (246.5 vs. 195.3, *p* = 0.013).

When considering SRH, those with higher SRH spent significantly more time in vigorous physical activity (11.5 vs. 8.7, *p* = 0.047), whereas residents with low self-rated health reported more total minutes/day spent sitting (708.5 vs. 616.0, *p* = 0.009) and more sitting time during weekend days (256.6 vs. 211.8, *p* = 0.023).

### 3.3. Association Between Physical Activity, Sedentary Behavior, Well-Being, and Self-Rated Health

Logistic regression analysis (Table 1) revealed a significant association between total daily hours spent in any physical activity level and well-being (aOR = 1.29, 95% CI = 1.02–1.63). The association between total walking hours was significantly associated with well-being [aOR = 1.42, (95% CI = 1.01–2.01)]. In contrast, vigorous physical activity was statistically significantly associated with higher well-being (aOR = 2.19, 95% CI = 1.11–4.35).

Total hours spent sitting (aOR = 0.94, 95% CI = 0.91–0.98) and sitting on weekdays (aOR = 0.93, 95% CI = 0.87–1.0) or weekend days (aOR = 0.91, 95% CI = 0.85–0.97) were strongly associated with low well-being. Time spent sitting is also associated with low self-rated health, either for total hours (aOR = 0.95, 95% CI = 0.91–0.99) or hours on weekdays (aOR = 0.92, 95% CI = 0.85–1.0).

### 3.4. Additional Analyses

**Figure 4:**
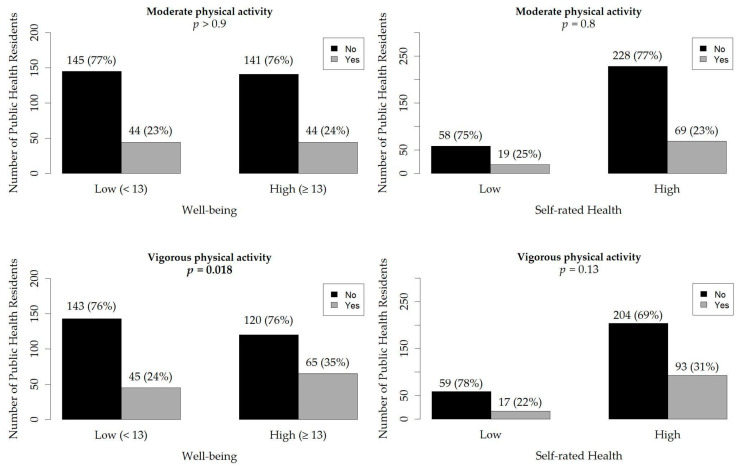
Bar charts with the number of public health residents who participated in this study and the related percentages, grouped by well-being and SRH.

According to WHO guidelines, 24% of PHRs met the recommended weekly amount of moderate physical activity, whereas 29% met the recommended weekly time dedicated to vigorous physical activity. Figure 4 shows the relation between PHRs’ adherence to WHO physical activity guidelines and either well-being or self-rated health. Among these associations, only vigorous physical activity was significantly related to higher well-being, with a higher percentage of high-well-being participants adhering to WHO guidelines (35% vs. 24%, *p* = 0.018). Similarly, Table 2 emphasizes that being adherent to the WHO recommendation to perform vigorous physical activity was significantly associated with high well-being (aOR = 1.65, 95% CI = 1.05–2.61).

## 4. Discussion

### 4.1. Interpretation of Findings

In our study, we meticulously assessed physical activity through various dimensions, including the total amount of hours of physical activity per day, hours spent walking, engaging in moderate and intense physical activities, and total sitting hours, along with a separate analysis for sitting hours during weekdays and weekend days. This comprehensive approach allowed us to capture a detailed picture of the residents’ physical activity patterns and sedentary behaviors.

Our results revealed statistically significant associations that provide a nuanced understanding of the impact of physical activity and sedentary behavior on self-rated health and well-being among medical doctor residents during the COVID-19 pandemic. Specifically, we found that the total amount of physical activity hours per day, the amount of walking per day, and the amount of vigorous physical activity hours per day were positively associated with better self-rated health and well-being scores. These findings suggest that not just any physical activity, but particularly walking and vigorous activities, contribute significantly to improving mental health outcomes in this population.

Conversely, our analysis also brought to light the negative implications of sedentary behavior on self-rated health and well-being. A statistically significant association was observed between the total number of sitting hours, including specific assessments of sitting hours during weekdays and weekend days, and lower well-being scores. This highlights the detrimental effects of prolonged sedentary behavior, which is particularly relevant in the context of the pandemic, where work-from-home arrangements and lockdown measures may have inadvertently increased sedentary time. This differential impact underscores the importance of not only promoting physical activity among medical residents but also addressing and mitigating sedentary behaviors as part of comprehensive mental health and well-being strategies during challenging times.

Our findings resonate with the broader body of research highlighting the positive impact of physical activity on mental health. For instance, a systematic review by Gianfredi et al. found that physical activity was beneficial in reducing depression among the general population [10]. Moreover, when assessing the association between daily patterns of physical activity, a recent systematic review found an association between lower physical activity during the morning, higher physical activity late in the evening (night), and depression or depressive symptoms [15]. However, our study extends this understanding to a specific, high-stress professional group—medical doctor residents—during an unprecedented global health crisis, thereby filling a crucial gap in the literature.

Some previous studies found a statistically significant association between light-intensity physical activity and mental health, specifically depression [13]. However, the results are discordant, with some other previous studies that did not find a clear dose–response relationship between the intensity of physical activity and mental health outcomes [36]. Our study suggests a more pronounced benefit of higher physical activity levels and better mental health outcomes. This discrepancy may be due to differences in study populations, measurement tools, or the unique stressors faced by our participants during the COVID-19 pandemic, which might make the benefits of physical activity more evident. Moreover, differences might also be due to the type of mental health outcome assessed. Previous evidence mainly focused on depression, depressive symptoms, and anxiety [37]. Furthermore, while previous research has often focused on the general population [38], our study provides valuable insights into the mental health of healthcare professionals, a group under considerable stress yet crucial to pandemic response efforts. This focus is particularly relevant given the increasing recognition of the importance of healthcare workers’ mental health for both their health and well-being and the quality of care they provide [39].

### 4.2. Potential Biological Mechanisms

The relationship between physical activity, sedentary behavior, and well-being can be explained through various biological mechanisms. Physical activity is known to stimulate neurochemical changes in the brain that contribute to improved mental health. Specifically, engaging in physical activities leads to the release of endorphins [40]. These biochemical changes are associated with feelings of euphoria and general well-being. Additionally, physical activity enhances the secretion of neurotransmitters like serotonin and dopamine, which play crucial roles in mood regulation and the prevention of depression and anxiety [41]. Physical activity also influences the hypothalamic–pituitary–adrenal (HPA) axis, reducing the body’s stress response. By moderating the release of cortisol, a stress hormone, regular physical activity can mitigate the physiological effects of stress, leading to improved mental health outcomes [42]. Moreover, lifelong physical activity might improve cardiorespiratory fitness, which has been associated with a lower risk of depression [43,44]. Conversely, sedentary behavior, characterized by prolonged periods of sitting or inactivity, has been linked to detrimental effects on mental health through several biological pathways [45]. Sedentary lifestyles can lead to alterations in brain structure and function, particularly in regions associated with mood regulation, such as the hippocampus [46]. These changes may increase susceptibility to stress and mental health disorders. Additionally, sedentary behavior disrupts metabolic health, which is closely tied to mental health; for instance, it can lead to inflammation and insulin resistance, both of which have been associated with depressive symptoms [47]. Moreover, sedentary behavior can exacerbate the negative impact of stress on the body. Extended periods of inactivity may enhance the sensitivity of the HPA axis to stress, leading to elevated cortisol levels and increased stress reactivity [48]. This heightened stress response can further impair mental health, creating a feedback loop that exacerbates the effects of both stress and sedentary behavior on well-being. The interplay between physical activity and sedentary behavior underscores the complex relationship between lifestyle factors and mental health. While physical activity acts to enhance mental health through positive neurochemical changes and stress reduction, sedentary behavior can counter these benefits through negative impacts on brain function, metabolic health, and stress reactivity. Understanding these mechanisms is crucial for developing targeted interventions that promote physical activity and reduce sedentary behavior to improve the mental health and well-being of medical doctor residents during the COVID-19 pandemic and beyond.

### 4.3. Implications for Public Health Policies

The findings of this study underscore the critical need for public health policies to address both physical activity and sedentary behavior among healthcare professionals, particularly during times of crisis such as the COVID-19 pandemic. Given the significant association between these lifestyle factors and well-being, interventions that encourage physical activity and reduce sedentary time could have substantial benefits for mental health.

Public health strategies could include the development and promotion of workplace wellness programs that offer structured opportunities for physical activity, such as group exercises, walking meetings, and stretch breaks during work hours [49]. Additionally, creating environments that discourage prolonged sitting, through the provision of standing desks and the promotion of regular breaks to stand or walk, could help mitigate the negative impacts of sedentary behavior.

Moreover, policy initiatives could focus on educating healthcare professionals about the mental health benefits of physical activity and the risks associated with sedentary behavior [50]. This could involve incorporating physical and mental wellness education into medical training curricula and offering ongoing support and resources for maintaining an active lifestyle amid the demands of the healthcare profession [51].

Thanks to the dissemination of the results of the PHRASI study through the Medical Residents’ Council of the Italian Society of Hygiene, Preventive Medicine, and Public Health, all these goals could be achieved starting from the settings of the Italian residency schools of public health. The Council is part of the Italian Society of Public Health (SItI). Thanks to SItI, the Residents’ Council works directly with university professors across the country and represents a crucial stakeholder in decision-making regarding training and education. The Council is producing a detailed report on the outcomes of the PHRASI study aimed at professors and directors of residency schools in order to build a better work environment for public health residents and to allow policymakers and the directors of residency programs to support the enhancement of physical and psychological well-being among medical residents. This effort could also provide evidence to support the upcoming national reform of medical residency schools.

### 4.4. Considerations for Future Research

Future research should delve deeper into the specific types and durations of physical activity that are most effective for improving mental health among medical doctor residents, as well as the thresholds of sedentary behavior that contribute to negative mental health outcomes. Longitudinal studies are needed to understand the causal relationships between physical activity, sedentary behavior, and well-being, including how these relationships may change over time or in response to interventions.

Additionally, investigating the impact of tailored interventions designed to reduce sedentary behavior and increase physical activity within the healthcare setting could provide valuable insights into effective strategies for improving mental health among medical professionals. This research should also consider the barriers and facilitators to engaging in physical activity and reducing sedentary time, including work schedules, access to facilities, and individual preferences.

Exploring the role of technology, such as wearable fitness trackers and mobile health apps, in promoting physical activity and reducing sedentary behavior among healthcare professionals could also offer innovative approaches to supporting their mental health and well-being. Furthermore, studies examining the interplay between physical activity, sedentary behavior, social support, and work–life balance could provide a more comprehensive understanding of the factors influencing mental health among medical doctor residents.

The COVID-19 pandemic was a determinant in the increase in sedentary behavior [52,53], especially among PHRs and other healthcare workers. Several reasons can explain this. The excessive workload due to both the lack of specialists in the Italian healthcare system and the dramatic increase in infected subjects could have led PHRs to have a reduction in available hours for physical activity [18,54]. Moreover, the restrictions for preventing the SARS-CoV-2 spread had determined the temporary closure of gyms and pools, as well as other open public spaces such as parks and green areas. Therefore, it should not be surprising that PHRs in this study had spent more hours sitting than doing physical activity. Considering the importance of physical activity for health, several interventions and simulations should be planned to promote any level of physical activity even in the context of possible future pandemic scenarios, as the evidence already reported [52,53]. As reported above, technological devices can help PHRs monitor the number of hours spent doing physical activity, the number of steps during walking or running, or the number of physical exercises [53,55]. Moreover, an implementation could also include the break times for physical activity during work and the monitoring of the number of active and inactive PHRs in the workplace to encourage physical activity during the break time [53].

The PHRASI study could serve as a foundation for future research endeavors encompassing residents from diverse medical specialties and as a national benchmark for comparing studies on healthcare workers’ well-being that involve medical residents. The Participatory Action Research (PAR) approach used in this study, the structure of the survey, the cooperation among local representatives of the Medical Residents’ Council from different universities, and the dissemination plan of the survey could inspire other residents’ research groups that want to create a nationwide study focusing on residents, even if exploring a different dimension than well-being.

### 4.5. Strengths and Limitations

One of the key strengths of this study is the adoption of instruments (IPAQ and WHO-5 Well-being Index) to measure physical activity and well-being widely used in the literature. While enhancing the internal consistency of the results and the reliability of the findings, the use of these tools also increases comparability with other studies’ results. Moreover, we captured a detailed picture of the residents’ physical activity patterns and sedentary behaviors through a comprehensive approach in various dimensions. Furthermore, we adopted statistical techniques, like logistic regression analysis, to explore the association between physical activity levels, well-being, and self-rated health, adjusted for sex and age. Additionally, the use of a large and nationally representative sample of PHRs, spanning all the course years of residency, increases the generalizability of the results to the broader population of medical doctor residents.

Nevertheless, our results should be viewed with some limitations that warrant consideration. Firstly, the cross-sectional design precludes inferring the direction of causality or temporal ordering, making it unclear whether higher levels of physical activity lead to better well-being or if individuals with better well-being are more inclined to engage in physical activity. Still, it is difficult to establish whether the absence of physical activity is a risk factor or a consequence of negative mental health outcomes because individuals with mental health disorders tend to have more sedentary behavior compared with others.

Finally, the study is vulnerable to recall bias because we used self-reported data rather than more accurate tracker-based measurements and participants may overestimate their levels of physical activity and well-being [56]. To further explore the relationships between physical activity, sedentary behavior, and well-being and to be able to ascertain causality and assess the temporality of the association at the symptom level, future longitudinal studies and objectively measured physical activity methods are needed.

## 5. Conclusions

This study illuminated the significant association between physical activity, sedentary behavior, and well-being among medical doctor residents during the COVID-19 pandemic. Our findings underscore the dual importance of promoting physical activity and mitigating sedentary behavior to support mental health in this high-stress population. Physical activity, particularly walking and intense activities, emerged as a beneficial factor for enhancing well-being, while sedentary behavior was associated with diminished mental health outcomes.

## Figures and Tables

**Table 1:** Logistic regression analysis adjusted for sex and age to explore the association between physical activity levels, sedentary behavior, well-being (WHO5), and self-rated health (SRH). Statistically significant associations are reported in bold.

	High Well-Being (WHO-5 ≥ 13)				High SRH			
Predictive Variables	aOR ^1^	95% CI		*p*	aOR ^1^	95% CI		*p*
Total PA (hours/day)	1.29	1.02	1.63	**0.032**	1.12	0.84	1.50	0.441
Walking (hours/day)	1.42	1.01	2.01	**0.046**	1.35	0.85	2.14	0.201
Moderate PA (hours/day)	0.97	0.64	1.45	0.874	0.99	0.60	1.63	0.976
Vigorous PA (hours/day)	2.19	1.11	4.35	**0.024**	1.46	0.61	3.52	0.397
Sitting total (hours/day)	0.94	0.91	0.98	**0.005**	0.95	0.90	0.99	**0.022**
Sitting at weekdays (hours/day)	0.93	0.87	1.0	**0.040**	0.92	0.85	1.0	**0.042**
Sitting on weekend (hours/day)	0.91	0.85	0.97	**0.007**	0.93	0.86	1.00	0.060

^1^ adjusted for sex and age; PA: physical activity; aOR: adjusted odds ratio; 95%CI: 95% confidence interval; WHO-5: 5-item Well-being Index.

**Table 2:** Logistic regression analysis adjusted for sex and age to explore the association between moderate and vigorous physical activity levels according to the WHO guidelines, well-being (WHO5), and self-rated health (SRH). The statistically significant *p*-value is shown in bold.

	High Well-Being (WHO-5 ≥ 13)				High SRH			
Predictive Variables	aOR ^1^	95% CI		*p*	aOR ^1^	95% CI		*p*
Moderate physical activity according to the WHO guidelines	1.03	0.64	1.67	0.889	0.94	0.52	1.69	0.835
Vigorous physical activity according to the WHO guidelines	1.65	1.05	2.61	**0.031**	1.54	0.85	2.82	0.155

^1^ adjusted for sex and age.

## Data Availability

Data are available upon request.

## Supplementary Materials

The following supporting information can be downloaded at: https://www.mdpi.com/article/10.3390/sports12120332/s1, Table S1: Summary of socio-demographic characteristics and physical activity levels of PHRs.
