# Supplementary material for: Physical Activity and Sedentary Behavior on Well-Being and Self-Rated Health of Italian Public Health Medical Residents During the COVID-19 Pandemic: The PHRASI Study
Source: Sports (Basel). 2024 Dec 2;12(12):332. doi: 10.3390/sports12120332 (PMC11679603; doi:10.3390/sports12120332)
Supplement: Supplementary file 1 [file sports-12-00332-s001.zip › sports-3118491-supplementary.pdf]

**Table S1.** Summary of sociodemographic characteristics and physical activity levels of PHRs.

| Characteristics of PHRs | N = 379[1] |
|---|---|
| **Sociodemographic variables** | |
| Age | 31.6 (4.5) |
| Sex | |
| Female | 219 (58%) |
| Male | 160 (42%) |
| Residence region | |
| North | 157 (41%) |
| Center | 96 (25%) |
| South and Islands | 126 (33%) |
| Work region | |
| North | 178 (47%) |
| Center | 113 (30%) |
| South and Islands | 88 (23%) |
| Cohabitation | |
| With others | 281 (74%) |
| Alone | 98 (26%) |
| In a stable relationship | |
| No | 103 (27%) |
| Yes | 276 (73%) |
| 5-item Well-being Index (WHO-5) | |

| | |
|---|---|
| Low (< 13) | 191 (50%) |
| High (≥ 13) | 188 (50%) |
| Self-rated health (SRH) | |
| Low | 79 (21%) |
| High | 300 (79%) |
| **Physical activity levels variables** | |
| Total PA (minutes/day) | 63.3 (60.5) |
| Walking (minutes/day) | 36.5 (37.2) |
| Moderate PA (minutes/day) | 16.6 (30.2) |
| Vigorous PA (minutes/day) | 10.9 (20.6) |
| **Sedentary behavior variables** | |
| Sitting total (minutes/day) | 635.3 (318.5) |
| Sitting on weekdays (minutes/day) | 414.1 (183.8) |
| Sitting at weekend (minutes/day) | 221.1 (188.0) |

[1] n (%); Mean (SD); PHRs: Public Health Residents; PA: physical activity
